# The State of Starch/Hydroxyapatite Composite Scaffold in Bone Tissue Engineering with Consideration for Dielectric Measurement as an Alternative Characterization Technique

**DOI:** 10.3390/ma14081960

**Published:** 2021-04-14

**Authors:** Mohd Riza Mohd Roslan, Nadhiya Liyana Mohd Kamal, Muhammad Farid Abdul Khalid, Nashrul Fazli Mohd Nasir, Ee Meng Cheng, Chong You Beh, Joo Shun Tan, Mohd Shamzi Mohamed

**Affiliations:** 1 Biomedical Electronic Engineering Program, School of Mechatronic Engineering, Universiti Malaysia Perlis, Arau 02600, Perlis, Malaysia; riza.roslan91@gmail.com (M.R.M.R.); nashrul@unimap.edu.my (N.F.M.N.); emcheng@unimap.edu.my (E.M.C.); neilbeh@hotmail.com (C.Y.B.); 2 Malaysian Institute of Aviation Technology, Universiti Kuala Lumpur, Dengkil 43800, Selangor, Malaysia; nadhiyaliyanamk@unikl.edu.my; 3 Faculty of Electrical Engineering, Microwave Research Institute (MRI), Universiti Teknologi MARA (UiTM), Shah Alam 40450, Selangor, Malaysia; mfarid044@uitm.edu.my; 4 Sports Engineering Research Centre (SERC), Universiti Malaysia Perlis, Arau 02600, Perlis, Malaysia; 5 Bioprocess Technology, School of Industrial Technology, Universiti Sains Malaysia, Gelugor 11800, Pulau Pinang, Malaysia; jooshun@usm.my; 6 Bioprocessing and Biomanufacturing Research Centre, Universiti Putra Malaysia (UPM), Serdang 43400, Selangor, Malaysia; 7 Department of Bioprocess Technology, Universiti Putra Malaysia (UPM), Serdang 43400, Selangor, Malaysia

**Keywords:** scaffold, bone tissue engineering, starch, dielectric properties, hydroxyapatite, free space measurement

## Abstract

Hydroxyapatite (HA) has been widely used as a scaffold in tissue engineering. HA possesses high mechanical stress and exhibits particularly excellent biocompatibility owing to its similarity to natural bone. Nonetheless, this ceramic scaffold has limited applications due to its apparent brittleness. Therefore, this had presented some difficulties when shaping implants out of HA and for sustaining a high mechanical load. Fortunately, these drawbacks can be improved by combining HA with other biomaterials. Starch was heavily considered for biomedical device applications in favor of its low cost, wide availability, and biocompatibility properties that complement HA. This review provides an insight into starch/HA composites used in the fabrication of bone tissue scaffolds and numerous factors that influence the scaffold properties. Moreover, an alternative characterization of scaffolds via dielectric and free space measurement as a potential contactless and nondestructive measurement method is also highlighted.

## 1. Introduction

Tissue engineering revolves around exploiting biological and engineering fundamentals to collocate cells and scaffold materials in assisting tissue growth and recovery process. It is favorably viewed as a feasible method to overcome transplantation issues due to inadequacies alluded to donor tissues or organs [1]. The success of tissue engineering depends on how the technique addresses multiple challenges in the form of the cell technology field, wherein the aspects that need to be highlighted include cell sourcing, cell function manipulation, and the effectiveness of stem cell technology. The challenges also encompass construction technology, which is closely associated with designation, tissue engineering construction and delivery transports, as well as manufacturing technology that is customized to suit the clinical needs and acceptance by the body in terms of immune acceptance [2]. It is opined here that natural biological implementation may manage some of the tissue engineering challenges in providing immense success for tissue and organ replacement, maintenance, and reparation.

**Figure 1:**
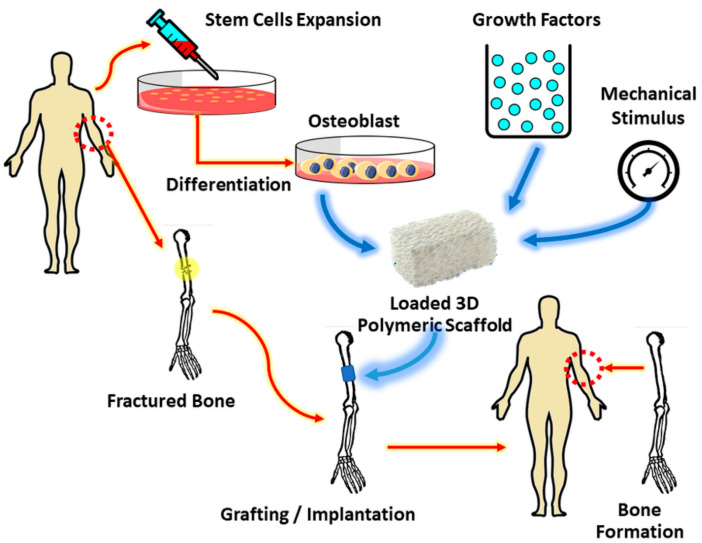
Bone tissue scaffold implantation.

Central to tissue engineering is the restoration of failing tissue and organ by a biological substitute through cell seeding and proliferation on a temporary scaffold to promote tissue growth and remodeling [3]. The types of cells for tissue engineering or organ substitution purposes can either be autologous (cells from the host itself), which exhibit no immune issues, or allogeneic (sourced from other donors), which can pose immune issues. In addition, the cells could even be xenogeneic (cells of other species) in which the recipient may be confronted with immune issues and virus transmission [4]. Xenogeneic technique interestingly had developed to patient-derived tumor xenograft (PDX) models for cancer research. This model could preserve the histology and genetic characteristics of the donor tumor, and as a consequence, it is favorable for preclinic drug evaluation, biomarker identification, biological studies and personalized medicine approach [5]. At present, bone repair uses regenerative treatment options, and scaffolds play a crucial role in bone tissue engineering. The scaffold material should ideally be biocompatible and biodegradable with a highly porous microstructure to accommodate cell attachment, proliferation, and growth stimulation [6]. For instance, the highly porous poly-(para-phenylene) bone implants demonstrated a favorable adherence site for mouse preosteoblasts cells (MC3T3-E1), consequently leading to better cell proliferation [7]. Figure 1 shows the sequence of bone tissue scaffold implantation procedures.

There are several approaches in treating the diseased or lost tissue in patients, such as via in situ regeneration whereby the external stimuli or specific scaffolds induce the tissue formation and stimulation of own cells from the body, leading to local tissue regeneration [8]. Another approach is via freshly isolated or cultured cells implantation, as this will be carried out by direct injection of cells or small cellular aggregates either from donor [9] or patient [10] onto the damaged or lost region without involving the degradable scaffold. Moreover, treatment could also be done through in vitro growth of three-dimensional (3D) tissue from autologous cells within the scaffold and then proceeding with the implantation procedure upon maturity [11]. In the latter category, utilization of autologous cells for bone reconstructions would entail an augmentation of the local host cells and transplantation of cells.

The augmentation procedure can be further branched into membrane techniques, biophysical stimuli, and biological stimuli. The membrane technique is based on the guided bone regeneration principle (GBR), in which the deployment of the resorbable membrane creates a barrier, separating the bone tissue from the ingrowth of soft tissue, thus creating an unrestrained space that permits the growth of a new bone. This type of reconstruction is generally used to rectify maxilla and mandible structure in maxillofacial surgery [12]. GBR strongly depends on the defect size and geometry, within which lies some of its limitations.

On the other hand, biophysical stimuli refer to inducement by mechanical and electrical sensations as bone formation regulators. Various clinical trials have demonstrated the efficacy of exposure to electromagnetic field (inductive coupling, capacitive coupling, and composite) and mechanical stimulation (distraction osteogenesis, low-intensity pulsed ultrasound, fracture activation) in hastening the bone healing process, leading to several clinically approved practices by relevant authorities [13].

Biological stimuli are attributed to signaling molecule cytokines involved in intracellular communication activity control and immunological reaction direction. Specific to bone construction applications, the cytokines in question can be further distinguished as a group of growth factors (GF), contributing to the effect that can be viewed in the context of the growth factor network. Chief among the growth factors is the superfamily of transforming growth factor-beta (TGF-*β*) with its three isoforms, namely TGF-β1, TGF-β2, and TGF-β3. These isoforms are crucial for bone tissue cell proliferation, differentiation, and remodeling processes. TGF-*β* is in consolidation with other proinflammatory cytokines, GFs, and angiogenic factors, i.e., fibroblast growth factors (FGF1 and FGF2), platelet-derived growth factor (PDGF), insulin-like growth factors (IGF-1 and IGF-2), bone morphogenetic proteins (BMP) family, and extracellular non-collagenous bone matrix proteins, namely osteonectin (OSN, SPARC), osteocalcin (BGLAP), and osteopontin (OPN, SPP1), all, of which are synthesized during distraction osteogenesis [14].

Scaffolds can be categorized based on their composition, external geometry, macro and microstructure, interconnectivity, surface per volume ratio, mechanical capability, degradation, and chemical properties. Aforementioned, scaffolds are templates for cells, and they grant the surrounding tissue ingrowth after implantation. The scaffold architecture may influence cell parameters, such as cell viability, migration and differentiation, and the substituted tissue composition. Loads gained at the implantation site would be retrieved by bone tissue scaffolds and delivered to the surrounding tissue, and thus the bone tissue scaffold is required to be mechanically competent to absorb the load after implantation [15]. According to Ahn et al., the biomechanical properties of poly-(para-phenylene) (PPP) bone implants were evaluated based on finite element modeling. From the finite element model, upon the stress loading, the stress dissipation is uniformly distributed onto the porous PPP. The results suggest that the porous structure of PPP is capable of minimizing stress shielding. The enhancement in the biomechanical feature is mainly contributed by the mechanical interlocking between the interface of the bone and the porous implant [7]. Previously, nondestructive mechanical analyses were performed by computed microtomography (micro-CT) to evaluate the internal structure of the materials and the performance of the bone scaffolds [16]. The microstructure scaffold defects could also be closely examined through the finite element mathematical modeling as studied by Naghieh et al. [17]. Here, the effect of post-heating on the elastic modulus and compression test of scaffold samples was computed via numerical analysis, which observed its microstructural performance. Recognition in microstructural imperfection is crucial as it could alter the mechanical criteria of porous materials and their cellular lattice structures. Another work on the mathematical modeling in bone tissue scaffold was also implemented by Avilov et al. [18], wherein the stress–strain of the lower jaw prostheses that consider the geometry, properties of bone tissue and mastication activity of patients was calculated. Thus, sufficient porosity is required to ensure bone and vascular ingrowth concurrently with tolerable mechanical properties for load-bearing [16]. Hollister [19] underlined the significance of scaffold materials and porous structural designs that fall in the region of 10 µm to 100 µm to manifest temporary mechanical function, preserve tissue volume, and deliver necessary biofactors (stem cells, genes and proteins) for stimulating the tissue repair. To achieve these goals, the hierarchical porous structure of scaffolds must be altered to suit the desired mechanical strength and mass transport. Therefore, porous structures should ensure cell migration occurs while encouraging the transportation of nutrients and cell attachment. Meanwhile, scaffolds must be mechanically strong to maintain their structural integrity during cell culture [20].

The extracellular matrices (ECM) of bone tissue are composed of inorganic and organic phases. Hydroxyapatite (HA) is chemically and physically similar to the inorganic components of natural bones. It also has excellent biocompatibility, osteoconductivity, and bioactivity. These place HA as one of the best candidates for the inorganic phase of ECM. Additionally, HA has a Ca/P ratio that falls in the range of 1.50–1.67, which encourages bone regeneration [21]. HA by itself is brittle and difficult to shape, and thus biopolymer is usually added to enhance its strength, as proven in the previous studies [3,22]. The typical biopolymer for this purpose is collagen, which is relatively poor in its mechanical strength. However, there are a few ways to improve this shortcoming, such as cross-linking, gamma radiation, and carbodiimide addition [23]. Therefore, a biocompatible material with all criteria matching or surpassing collagen should be considered to fabricate an excellent bone tissue scaffold. The candidate biomaterial should preferably come from non-fossil or petroleum resources [24].

Gomes et al. [25] demonstrated that starch-based scaffolds supported attachment, proliferation, and differentiation of bone marrow stromal cells. Starch has been studied as one of the potential biomedical materials due to its low-cost, abundance in nature, excellent hydration, and high biodegradable property [26,27]. From the manufacturing point of view, starch is fascinating as it can be easily formed through conventional polymer processing techniques, such as extrusion, molding, thermoforming, and blowing [28]. The constraint of adopting starch relates to processing issues, low mechanical strength, and sensitivity to water. Several works to overcome these problems have been experimented with via additives and chemical modification. Previously, bone scaffolds were fabricated from a single material without assimilating with other types of biomaterial. Recently, natural or synthetic polymers were formulated with HA. One of the motivations was to add other types of biomaterial to improve porosity [29].

HA is the mineral form of calcium apatite with chemical formula as ${\mathrm{Ca}}_{10}{\left({\mathrm{PO}}_{4}\right)}_{6}{\mathrm{OH}}_{2}$. It is the principal inorganic biomineral phase of the human hard tissue found in teeth and bone to the tune of 60–70 wt.% [30]. Its crystal structure is a hexagonal cylinder, and each unit cell is made up of 44 atoms (10Ca^2+^, $6{\mathrm{PO}}_{4}^{3-},$ and 2OH^−^) formed by a tetrahedral arrangement of phosphate (${\mathrm{PO}}_{4}^{3-}$), which constitutes the skeleton of a unit cell [31]. HA crystal system belongs to a hexagonal space group of P6 3/m. The space group comprises six-fold of c-axis and is perpendicular to three equivalent a-axes at an angle of 120° to each other. The lattice parameters of a unit cell of HA are a = b = 0.9422 nm, and c = 0.688 nm, respectively [32]. Popular methods of HA synthesis included wet chemical precipitation, sol–gel method, hydrothermal method, and microwave irradiation method [33,34,35].

HA is a well-received bioactive material for biomedical applications in orthopedics and dentistry due to its various meritorious properties, such as excellent biocompatibility, bioactivity, and osteoconductivity [36]. HA has been implemented as a coating material for metallic biomaterials in the past decades [37]. Swain et al. [38] studied the HA-based scaffolds and showed that these scaffolds exhibited good bioactivity and bioresorbability during the in vitro assessment. As implants, in vivo and in vitro studies favorably indicated that synthetic HA could promote new cell differentiation and proliferation without causing any local and systemic toxicity or inflammatory responses [31]. Despite this, scaffold construction that combines biopolymer, such as starch with HA ceramic, is necessary to overcome the HA inherent material characteristics, whereby its hard but brittle nature severely limits its load-bearing applications and malleability into complex shapes and manipulation into defect specific sites [39].

Starch is the primary form of carbohydrate in plants. It can be sourced out relatively cheap due to its availability from diverse resources, such as roots (cassava, potatoes), crop seeds (rice, wheat, corns, peas), and plant stalks (sago) [40]. Starch content may vary between sources like grains (≈30–80%), legumes (≈25–50%), and tubers (≈60–90%) [41]. Starch consists of two polymers of D-glucose: linear amylose, which is essentially unbranched α[1 → 4] glycosidic linked glucan (20–30%), and a much larger, non-linear amylopectin (60–90%), which has chains of α[1 → 4] linked glucose arranged in a highly branched structure with α[1 → 6] branching links [42]. Native starch exists in the form of semi-crystalline granules with a complex hierarchical structure. Together, amylose and amylopectin make up 98–99% of these granules’ dry weight, while the remaining fractions comprise lipids, minerals, and phosphorus in the form of phosphates esterified to glucose hydroxyls. Starch granules differ in shape (polygonal, spherical, lenticular) and size (1–100 μm in diameter). These traits depend on the content, structure and organization of the amylose and amylopectin molecules, branching architecture of amylopectin, and degree of crystallinity [43]. Native starch extracted from plants cannot tolerate extreme processing conditions, such as high temperature, freeze-thaw cycles, strong acid and alkali treatment, and high shear rates [42,44]. Nevertheless, processes, such as plasticization of starch [45] and compositing it with other materials, e.g., halloysite nanotubes (HNTs), will further reinforce the mechanical, thermal, and swelling properties of starch, resulting in a porous matrix with a promising potential for biomedical applications [46].

## 2. Starch/Hydroxyapatite Composite Scaffold

Previous work on tissue engineering has shown that nano-HA can improve the function of the scaffold by providing a much larger surface area [47]. Still, HA-based ceramic scaffold performance in treating bone defect is limited by its brittleness. Another problem associated with HA is that its degradation rate is difficult to control [48], which has imposed challenges in determining the scaffold suitability for implantation. As one of the most abundant natural biopolymers, starch has been considered a component of the scaffold composites in tissue engineering due to its biodegradability and biocompatibility. Cytotoxicity analysis performed on the starch/HA scaffolds shows that the scaffold did not induce toxicity to mammalian cells [49]. The incorporation of starch could reduce the brittle nature of the HA scaffolds. This is due to the helical structure of amylose in starch, which formed an open network structure when it is stretched. This network comprises the hydrophilic exterior surface and hydrophobic interior cavity, which interacts with HA nanoparticles. This interaction would consequently create adhesive forces between the polymeric network and HA nanoparticles, thus improving the strength of the HA scaffolds via interlocking mechanisms [39,50].

In the latest study by Beh et al. [51], the scaffold made of corn starch and nanohydroxyapatite (n-HA) composite has a network of macropores (200–600 μm) and micropores (50–100 μm). It has a high degree of interconnectivity, suggesting that highly porous cornstarch/HA endowed with good mechanical properties can be a potential biomaterial for bone tissue engineering applications. The combination of starch and HA can influence the mechanical properties of scaffolds through pore size manipulation. Therefore, the scaffold must be designed to meet specific porosity requirements to facilitate cell attachment and migration, apart from having sufficient mechanical strength to support newly generated tissues. These porosity requirements include the size of pores, the interconnectivity of pores, and distribution. Table 1 lists a number of significant studies pertaining to starch/HA composite bone scaffold. On the other hand, Table 2 indicates the pore size required to support the regeneration of bone tissues [52].

**Figure 2:**
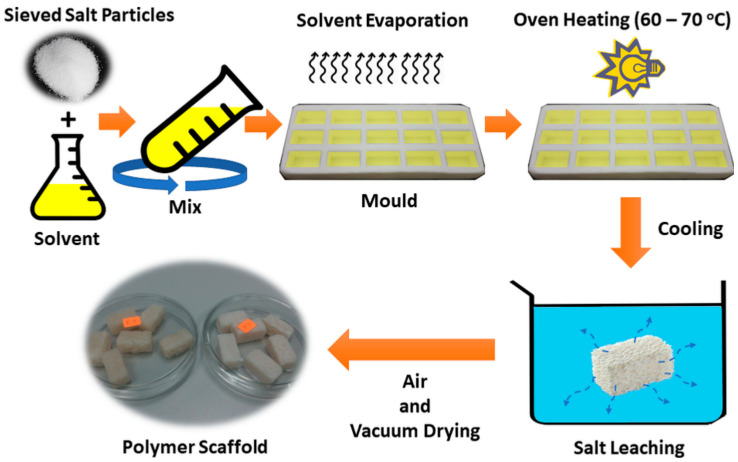
Schematic diagram for solvent casting and particulate leaching method.

Several factors affect the properties of the fabricated scaffold. This includes the processing methods, botanical origin of biopolymer, composition of biocomposite, and sintering temperature. Based on these factors, the fabrication of a scaffold can be optimized to meet the desired porosity and strength. Studies by Gomes et al. [60] and Tiwari et al. [61] had focused on the effects of different processing techniques on the structural properties of a scaffold. The techniques investigated included extrusion by using blowing agents, compression molding, solvent casting and evaporation, in situ polymerization method, and particulate leaching (the example procedure is shown in Figure 2). It was demonstrated that, although the morphology and the mechanical properties of the scaffold were tailored via different processing techniques, the biocompatible behavior of the starch-based scaffold was not affected.

The scaffolds fabricated via extrusion through the use of a blowing agent based on carboxylic acid by Gomes et al. [60] have been shown to produce pore sizes of 50–300 μm and porosity of 40–50%. An improvement in pore interconnectivity and pore size in the range of 100–500 μm was achieved when a blowing agent based on citric acid was used. Scaffold with a pore size of 10–500 μm and a porosity of 50% was also reported when fabricated via compression molding and particle leaching technique. Through this technique, the porosity of the scaffold was controllable by modifying the amount and size of the particle used. The authors’ SEM images showed that solvent casting and particle leaching technique eventually resulted in the best pore interconnectivity compared to the earlier mentioned techniques, with a pore size ranging from 50–300 μm and porosity of 60%. This processing technique also allowed accurate control of desired porous structural properties by controlling the particles’ amount, shape, and size. Larger scaffold porosity would allow more spaces for new cell growth, which was much more desirable.

Besides conventional melt-based processing techniques, advanced processing technologies, such as rapid prototyping [52], can also produce scaffolds with such accurate control of the scaffold properties at macro and micro scales. This is done with computer-aided-design (CAD) modeling tools and 3D printing of the scaffold. Sears et al. [62] aimed to develop printing tools and suitable materials as bio-ink that might fulfill the requirement of a biocompatible scaffold. It was demonstrated by Sobral et al. [63] that the pore size gradient of scaffolds fabricated via rapid prototyping could increase the seeding efficiency from approximately 35% to 70%. Electrospinning is another advanced technology for scaffold fabrication, particularly for a design that involves nanofibers. The enhanced cellular activity was achieved by employing this technique, which was attributed to the enlargement of the scaffold surface area [64]. Electrospun nanofiber scaffolds based on HA and native cellulose had exhibited porosity in the range of 50 to 500 nm. The addition of nano-HA displayed an increment in the average fiber diameter [65]. Overall, these advanced technologies were proven to impart better control over the scaffold morphology and thus the functionalities associated with it as well.

Other than fabrication techniques, the amount of biopolymer added during fabrication also affects the scaffold properties. By varying the amount of potato starch as the biopolymer in scaffold formulation, Ahmed et al. [66] reported that SEM images taken from the fabricated scaffold showed an increase in porosity from 28% to 53% as the starch amount was increased from 10 vol% to 30 vol% (percentage of starch in the composite mixture). An increase in the starch content also changed the pore shape from a spherical-like shape (in low starch content) to an irregular shape (in high starch content). The compressive strength increased along with the increased amount of starch addition of up to 30 vol% but decreased thereafter. The increase in compressive strength relative to the additional amount of starch resulted from the binding effect among starch granules [67]. Further addition of starch beyond 30 vol% had weakened the compressive strength as more voids (due to higher porosity) contributed to reducing porous structure strength.

In addition to starch concentration, the work conducted by Ahmed et al. [67] also revealed the heat treatment effect on starch-loaded HA scaffold. An experiment was conducted whereby the HA was treated at 1100 °C and then compared to the as-received HA sample. It was discovered that with the heat-treated HA, the amount of solid loading when using native corn starch could reach up to 59 vol.% as compared to only 14 vol.% for the non-heat-treated HA. Beyond the limits of solid loading, the produced slurry appeared to have a paste-like consistency. Achieving a higher limit of solid loading would allow for exploring the advantages of higher porosity and mechanical strength from increased starch content. For instance, mechanical analysis in terms of scaffolds’ stiffness was performed to examine the structural integrity after 14 weeks implanted in the nude mouse model [68,69]. Typically, the most common mechanical analysis done on bone scaffolds is compressive stress [70,71]. Beh et al. [51] have shown that the compressive strength of porous 3D HAp samples increases in proportion to corn starch.

Sintering temperature may also affect the properties of scaffolds made from calcined HA and potato starch, as observed by Ahmed et al. [57]. It was demonstrated that the increase in sintering temperature had resulted in porosity decrement. For instance, at 30 vol% amount of starch, the resulted porosity was about 57%, 53%, and 50%, corresponding to the sintering temperature of 1250 °C, 1300 °C, and 1350 °C, respectively.

Several starches from different botanical origins were used in the scaffold fabrication, in which NaCl was used as the porogen [72,73,74]. In these studies, a scaffold that possessed high porosity and high water uptake abilities could be achieved by increasing the starch concentration to a certain level. The botanical origins of starch used were “Balik Wangi”, a variety of fragrant rice, “Ubi Gadong”, or Indian three leaf yam (*Dioscorea hispida*), and brown rice. Scaffolds were fabricated using solvent casting and particulate leaching technique, and the effects of varying the amount of starch were investigated. Results obtained were in agreement with other earlier works, indicating the increase in porosity as the starch amount increased.

Although the experimental setup was generally similar between the work done by Mohd-Riza et al. [72], Hori et al. [73], and Mohd-Nasir et al. [74], the different botanical origins of starch resulted in different pore sizes, as revealed from their respective SEM images. The scaffolds fabricated using starch from “Balik Wangi” rice, “Ubi Gadong”, and brown rice had a pore size in the range of 10–400 μm, 80–600 μm and 138–1010 μm, respectively. Although data on compressive strength were not available in these studies, previous literature suggested that a different pore size range would result in the variation of compressive strength. Therefore, a correct selection of botanical starch origin has the potential to tailor the properties of the scaffold for the intended application. It can be inferred here that in a scaffold with a fixed amount of HA, the amount of the starch content added plays an important role in affecting the performance of the scaffold. On the other hand, manipulating the HA content also significantly altered the mechanical and porosity of bone scaffolds [22,75]. It was suggested by Chen et al. [76] that the diversity in grain size exerts several effects typically on chemical composition and macroporous structures of the biocomposite scaffold. In the bone scaffold itself, the grain size will definitely affect the protein adsorption as the larger grain size will provide an extra protein site that will promote cell adhesion and proliferation [77,78].

The amylose content in starch varied from different botanical origins. It was reported in the literature that starch with high amylose content might improve properties, such as tensile strength, elongation, and impact strength [79]. Koski et al. [54] studied the effect of amylose content on the mechanical properties of starch/HA bone scaffolds. The comparison was performed on the total amylose content in corn, potato and cassava starch, which showed that compressive strength was increased as the amylose content increased, as the amount had affected the physicochemical and functional properties of the scaffolds, such as the swelling capability and solubility. The amylose content of starch from banana, corn, and potato was reported to be between 17% and 24%, while starch from rice had amylose content between 15% and 35% [80]. The amylose content of sago reported by Misman et al. [81] was approximately 27%. The high amylose content in sago has made it a promising material for the fabrication of the HA–starch-based scaffold. Previous studies on scaffold based on sago starch and hydroxyapatite are limited. The study performed by Mustaffa et al. [82] had used sago and polyvinyl alcohol as a binder in the fabrication of HA and alumina composite. The effect of sintering temperature on the strength of the scaffold was the focus of the study. Here, sago was not treated as one of the main components in scaffold fabrication. Given the high amylose content of sago starch compared to other botanical origins of starch, it is worth exploring the potential of sago starch to produce scaffolds with desirable properties. Unfortunately, the brittle nature of starch alone has limited its application. Further adjustments, such as modification and blending with other polymers, are needed to overcome this issue.

## 3. Starch as Particulate Pore Former

Porous ceramics have been widely applied in filtration membranes [83] and catalyst support [84], apart from their application as bone tissue scaffolds for bone ingrowth and drug delivery systems. Porosity and pore interconnectivity is important criteria in bone tissue scaffolds because the interconnection of pores would enhance the nutritional supply. Therefore, this will be adequate for cell survival in the deeper area of the scaffold. This condition is directly affected by the scaffold macropore size, ratio, and morphology. Macropores with 100 µm in diameter size can execute the function of cellular and extracellular components of bone tissue and blood vessels. Meanwhile, pores that are larger than 200 µm in diameter would facilitate osteoconduction [52]. Moreover, the material porosity improves contact between host tissue and ceramic implants, which promotes better interface and reduces movability [85].

Furnishing macro-porosity in ceramic bodies requires the mixing of porogen and pore-forming agents during the manufacturing process. Theoretically, the porogen and pore-forming agents will be discarded through heating and dissolution. Subsequently, this will leave free spaces or voids in ceramic bodies known as pores [86]. Numerous porous ceramic applications crucially require definite control on porosity, pore size, pore shape, and pore space topology. Biological pore-forming agents may be ecologically advantageous and biocompatible. Few starch types were used, covering sizes that range from 5 µm (rice starch) to 50 µm (potato starch) and burning these starches at around 500 °C would create porosities in ceramic bodies [87]. Besides, starch addition in porous ceramic was driven by its gelling ability, mainly as a binder when immersed in water at 60 °C to 80 °C [88]. Xu et al. [89] had employed the corn starch consolidation method in aluminum titanate (${\mathrm{Al}}_{2}{\mathrm{TiO}}_{5}$)-mullite (M) ceramic to exploit the pores in ceramic. Based on the authors’ observation, the pore size was bigger as the corn starch percentage increased. Basically, the formation of the pores was due to the volatilization of corn starch. Pore sizes obtained were in the range of 10 µm to 15 µm. Experimentally, the 10% addition of corn starch achieved 54.7% of apparent porosity and 11.5 MPa flexure strength.

In another work, yttrium oxyorthosilicate ($Y_{2}{\mathrm{SiO}}_{5}$) ceramic was introduced with starch to create the porosity in ceramic. An increase in starch addition from 10 wt.% to 40 wt.% notably affected the reduction in ceramic porosity in the range of 70.4% to 38.3%, while the compressive strength ranged from 28.25 MPa to 1.43 MPa [90]. Si–O–C (“black glass”) was prepared from the foaming of polysiloxane and starch in other ceramic applications. The addition of starch improved ceramic porosity, whereby porosity obtained was 70% to 90% with compressive strength of 2 MPa to 16 MPa [91].

Similarly, starch as the pore generator was employed in scaffolds as studied by Hadisi et al. [59]. Theoretically, the formation of imine conjugation (Schiff base) between aldehyde from starch and amino groups in chitosan executes porosity in scaffolds. The imine formation displaces the water molecule, and this may increase the porosity and pore size during the freeze-drying process. Calcium phosphate granules were employed in the application of osseous fillers and drug carriers by Marques et al. [92]. In their report, HA and β-tricalcium phosphate (TCP) doped with strontium and magnesium were prepared via the precipitation method. When starch was employed as the pore-forming agent, Ozturk et al. [93] found that the porosity was interconnected in a perfect spherical shape.

Determining scaffold porosity through a conventional method, such as liquid displacement and volume change [93], is a destructive approach. Alternatively, nondestructive testing is mainly directed for hydrophilic-material-based scaffold, and thus porosity characterization of the scaffold can be performed by utilizing microwave measurement. Characterization of the scaffold, such as pore size via SEM, is comparatively costly and does not allow for real-time monitoring of the porous scaffold after implantation. Apart from this, the scaffolds’ interconnectivity and their overall porosity are quite impossible to be determined [94]. Due to this reason, Ahn et al. [7] proposed micro-CT analysis to measure the porous structure of the polyetheretherketone for orthopedic implants.

For the past few years, dielectric spectroscopy has been applied upon human and animal tissue as in vitro measurements through dielectric properties determination. For instance, the effect of cross-linking collagen against the dielectric properties was studied by Marzec et al. [95]. The value of dielectric measurement was found to be affected by the changes in collagen structures, mainly due to the release of the water molecule. Microwave is an electromagnetic wave with very short lengths and exceptionally sensitive to the dielectric property of materials. Microwave materials are extensively used in telecommunication, microwave electronics, radar, industrial microwave heating, and aerospace materials. It is important to characterize these materials for absorption, transmission, reflection, dielectric properties, and magnetic properties as a function of frequency. The relative to free space dielectric properties of a material is generally a complex parameter, whereby the real part indicates the material ability to store microwave energy, while the imaginary part indicates the material ability to absorb microwave energy [96].

**Figure 3:**
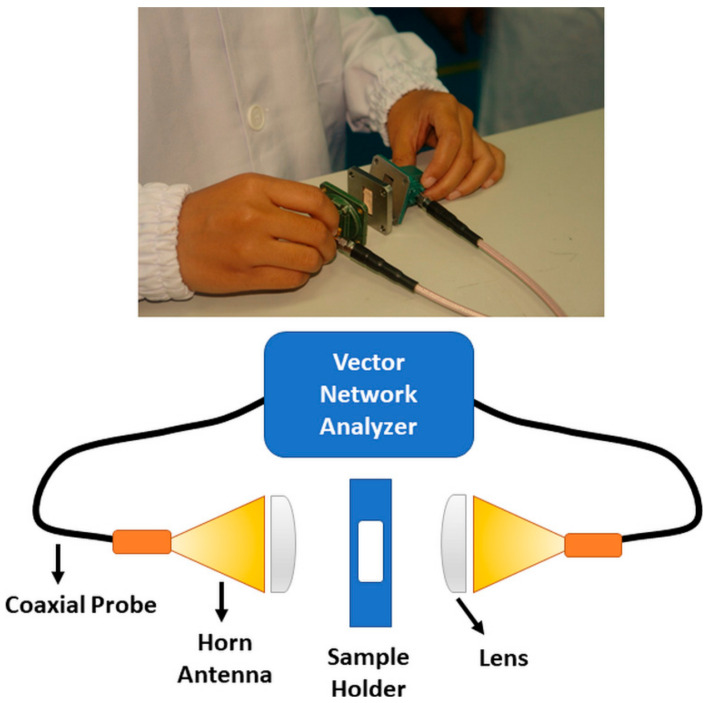
Vector network analyzer to extract the dielectric properties of a material.

Several techniques are available to determine the dielectric properties by using microwave measurement, which depends on factors, such as frequency of interest, desired accuracy, material form (either liquid or solid) and whether the sample can be tested under direct contact or otherwise [1]. Techniques for dielectric measurement in the microwave range include a coaxial probe, transmission line, free space, and resonant cavity. The coaxial probe is suitable for the measurement of materials in the form of liquid or semi-solid, and the measurement requires the probe to be in contact with the material. Measurement using transmission line, resonant cavity, and parallel plate imposes restrictions on the sample size and shape. So far, the free space measurement method is the only non-contacting method of all methods mentioned. Hence, it reduces the possibility of damaging the sample and leads to a more accurate dielectric measurement [2]. Figure 3 shows the free space measurement technique, which consists of a vector network analyzer to extract the dielectric properties of specific material. The dielectric measurement was performed using the parallel plate method, which is suitable only for low frequency. Although the contactless measurement approach can be achieved via some modification of the parallel plate method, it is not suitable for measuring the microwave range.

## 4. The Effect of Porosity in Ceramic over Microwave Dielectric Measurement

The dielectric measurement was widely applied in ceramic materials, focusing on the dielectric constants and losses. Dielectric loss is regularly characterized by imaginary part to the real part of permittivity and notated by tan δ. Losses are classified into two types, which are intrinsic and extrinsic. The intrinsic losses are dependent on the crystal structure, which is described as an interaction between the phonon system and the electric field. Extrinsic losses are related to imperfection in the crystal structure. These imperfections include impurities, microstructural defects, grain boundaries, porosity, microcracks, and random crystallite orientation [97]. Lanagan et al. [98] examined the effects of porosity and microstructure on dielectric properties upon titanium dioxide (TiO_2_) in rutile. Dielectric measurement was done in terms of the relative permittivity (ε_r_), loss tangent (tan δ), and temperature coefficient of resonant frequency (TCF). By focusing on the porosity, it was observed that the tan δ was greatly influenced by pore volume, while ε_r_ was less significant towards porosity.

A study by Zhao et al. [99] approached the dielectric measurement of boron nitride/silicon nitride (BN/Si_3_N_4_) ceramic by adding Y_2_O_3_–MgO_2_ additive powder to manipulate the porosity in ceramics. Introducing Y_2_O_3_–MgO_2_ additive powder seemed to increase the relative density of (BN/Si_3_N_4_) ceramic while the apparent porosity of ceramic decreases. Porosity and phase components greatly influenced the dielectric properties of ceramic. The effects were notated by Lichtenecker’s mixed logarithmic law. Basically, increment in Y_2_O_3_–MgO_2_ additive powder will reduce porosity, and this will consequently raise the dielectric constant (ɛ) and dielectric loss tangent (tan δ).

Pores are subjected to a decrease in ɛ and tan δ. It can be seen that the ɛ and tan δ of BN/${\mathrm{Si}}_{3}N_{4}$ ceramic had increased as the porosity decreased [99]. Dielectric measurement based on reflection and transmission was similarly applied in porous yttrium silicate (Y_2_SiO_5_) ceramics by Zhang et al. [90]. From their experiment, the ceramics were fabricated through the freeze casting method. The increase in solid content of the ceramics was from 15 vol.% to 30 vol.%, which decreased the porosity and pore channel size.

There have been few studies reported on the dielectric measurement of scaffolds [3,6,11,12]. In Lang et al. [12], the properties of chitosan/nano-hydroxyapatite composite were investigated using dielectric constant measurements in the frequency range of between 40 MHz and 110 MHz. It was revealed that the dielectric constant increased with the increasing concentration of nanoparticles. However, most of these works employed non-contactless measuring methods, such as a resonant cavity, transmission line using waveguide, dielectric probe, and parallel plate method.

Dielectric properties studies were further expanded on various starches, including tapioca, corn, wheat, rice, waxy maize, and Basmati rice [100,101]. As such, the quantification of dielectric properties via free-space measurement on the starch/HA scaffold in bone tissue engineering is a new area to explore. The measurement of bone tissue scaffolds’ porosity based on dielectric spectroscopy is still at a novel stage. This new research direction is currently undertaken by Razali et al. [102], Beh et al. [103], Roslan et al. [55] and Mohd Nasir et al. [104], concentrating on starch/HA scaffolds. Their research was focused on the correlation between the dielectric properties of bone scaffolds against the porosity, while other researchers delved more into the material dielectric properties. This new nondestructive alternative method seemingly offers a new approach in measuring the porosity of scaffold compared to the conventional method, such as the liquid displacement method. The application of dielectric measurement to determine the porosity of bone scaffolds is sensible for hydrophilic-material-based scaffolds. This is because the porosity measurement through the liquid displacement method that uses solvents, such as distilled water, will cause these types of scaffolds to rupture and swell, making such measurement difficult.

Studies by Razali et al. [22], Beh et al. [103], Roslan et al. [72] and Mohd Nasir et al. [74] involved the measurement of dielectric constant (ε^′^) and dielectric loss (ε^″^) of the starch/HA bone tissue scaffolds by using transmission line method at frequencies that ranged from 12.4 GHz to 18 GHz [55,104]. Here, the dielectric spectroscopy measurement applies to any porous composite scaffolds as the porous architecture could be quantified by respective ε′ and ε″ value. The corn starch/HA scaffolds exhibited low ε′ and negative ε″, which were influenced by the composites porous morphology and their crystalline features due to the various proportion of HA and corn starch applied [50,102,103]. This similar trend could also be seen in tapioca starch/HA scaffolds [104]. However, not all starch/HA composites would exhibit similar dielectric properties to be proportionate to the amount of starch added to the HA, as expected. Roslan et al. [55] found that the size and distribution of micropores of the scaffolds did not correspond to the increment of Bario rice starch added to the HA composites. Therefore, this phenomenon has verified the relation between physicochemical and dielectric properties of the porous composite. Thus, this discovery may initiate the basis of the nondestructive microwave evaluation test for porous composites.

## 5. Conclusions

The factors that improve the properties of a scaffold, particularly in terms of its structure, including using a larger amount of starch, sintering at a lower temperature, and using heat-treated hydroxyapatite. The use of starch with high amylose content could be the key for higher quality scaffolds produced from HA–starch composites. Additionally, the percentage of porosity and pore sizes of a scaffold to date are usually characterized by using costly, non-contactless, and destructive methods. Therefore, an alternative for scaffold characterization can be performed via microwave measurement to determine the dielectric properties of a particular scaffold. Furthermore, the correlation between dielectric properties and structural properties could be used as the initial work for future biomaterial-based scaffold characterization, perhaps by including the material mechanical properties and biocompatibility.

## Figures and Tables

**Table 1:** Research on starch/hydroxyapatite (HA) composite scaffold.

Technique	Pore Size/Porosity	Findings	Reference
Solvent casting and particulate leaching	Pores were formed with a size of 163 mm	Produce excellent mechanical strength The pore size in a range of 14 µm to 17 µm and dependent on the size of the salt used	[49]
Spin coating	Interconnecting porosity appeared	40 wt.% of HA incorporate with starch composite have tensile strength 303 ± 003 MPa, elongation 215 ± 55% and modulus 155 ± 02 MPa	[53]
Solvent casting and particulate leaching	Micropores size range from 622 µm to 966 µm, while macropores size range from 3683 µm to 5517 µm	The higher ratio of FTIR peak intensities between HA particles and starch may imply the higher bond strength	[50]
3D printing	Little microporosity suggesting the scaffold is fully dense	Compressive strength achieved is 1249 ± 022 MPa for 546 wt.% corn starch	[54]
Solvent casting and particulate leaching	Porosity obtained in range 163 µm to 282 µm	Measurement of porosity via dielectric spectroscopy via the value dielectric loss and dielectric constant air matrix	[55]
3D printing	Little microporosity suggesting the scaffold is fully dense	Higher starch loading was found to improve mechanical strength from 407 ± 066 MPa to 1035 ± 110 MPa	[39]
Freeze drying	Porosity up to 95% with pore size in the 80 µm to 292 µm range	Cellulose nanofibers and HA nanoparticles are incorporated into the cross-linked starch/PVA and formed bone scaffold. The scaffolds’ compressive properties were improved MTT assay measurement shows the scaffold has excellent cytocompatibility	[56]
Solvent casting	The highest porosity can be achieved up to 57% contained 30 wt.% potato starch sintered at 1250 °C	Pore size was positively affected by the starch amount and sintering temperature The higher amount of starch and lower sintering temperature leads to high retardation in compressive strength	[57]
Freeze drying	Pore size between 150 µm to 200 µm	Interconnected pore structure with semispherical and irregular shape Incorporation of starch HA into collagen HA sponges its elastic modulus	[58]
Electrospinning	Porosity after incorporation with silk fibroin nanofiber from 6027% to 6714%	Increasing the amount of silk fibroin nanofiber, the average pore size, porosity and swelling ratio decreased in starch HA composite High viability of osteoblast cells on the composite scaffold	[59]

**Table 2:** Pore size distribution for an ideal scaffold in bone tissue engineering application.

Pore Size (μm)	Biological Relevance
<1	Protein interaction and adsorption
1–20	Initial cell attachment
20–100	Cell proliferation, migration
100–1000	Cell growth and collateral bone growth
>1000	Essential for maintenance and programming

## Data Availability

No new data were created and analyzed in this study.
